# Evaluation of Phosphine Resistance in Populations of *Sitophilus oryzae*, *Oryzaephilus surinamensis* and *Rhyzopertha dominica* in the Czech Republic

**DOI:** 10.3390/insects13121162

**Published:** 2022-12-16

**Authors:** Radek Aulicky, Vaclav Stejskal, Barbora Frydova, Christos Athanassiou

**Affiliations:** 1 Crop Research Institute, Drnovska 507, 161 00 Prague, Czech Republic; 2 Laboratory of Entomology and Agricultural Zoology, Department of Agriculture, Crop Production and Rural Environment, University of Tessaly, Phytokou Str., 38446 Nea Ionia, Magnesia, Greece

**Keywords:** resistance, phosphine, stored-product Coleoptera, quick diagnostics, knockdown, rice weevil, lesser grain borer, saw-toothed grain beetle

## Abstract

**Simple Summary:**

Storage arthropods may invade and damage many types of stored commodities, both in developing and developed countries. The continuous worldwide usage of phosphine as a major fumigant for stored grain protection has led to the development of resistance by several major stored-product insect species. There are data on the occurrence of phosphine resistance from different pest species originating from multiple countries and geographical areas, especially in countries that are among the major grain producers in the world. However, the ongoing EU project (novIGRain) has recently enabled a resistance survey of the selected key species of stored-product pests in some EU countries, including the Czech Republic. The presented part of the survey in this publication included more than fifty field populations of the rice weevil, *Sitophilus oryzae*, the lesser grain borer, *Rhyzopertha dominica*, and the saw-toothed grain beetle, *Oryzaephilus surinamensis*, collected in Czech farm grain stores. It was found that the tested Czech populations of *O. surinamensis* have a relatively lower frequency and level of resistance than the populations of *S. oryzae* and *R. dominica*. Based on our results, the occurrence of resistance to phosphine in the Czech Republic is widespread and includes economically important species, which highlights the need for fumigation alternatives as well as the establishment of integrated resistance management programs.

**Abstract:**

Phosphine is globally the most widely adopted fumigant for the control of storage pests. Recently, an increase in the frequency of stored-product pest resistance has been observed with significant geographical and interspecific variations. In this context, there are available data for the occurrence of resistant populations from America, Asia, Africa, and Australia, but there are few data in the case of Europe. Therefore, the aim of this work was to evaluate phosphine efficacy in important beetle pests of stored products, i.e., *Sitophilus oryzae* (L.), *Oryzaephilus surinamensis* (L.), and *Rhyzopertha dominica* (F.) sampled from the Czech Republic, using a rapid diagnostic test that is based on the speed to knockdown after exposure. Apart from the standard laboratory populations, which were used as the controls, we tested 56 field populations of these three species, collected in Czech farm grain stores. The survey revealed that 57.1% of the tested field populations were classified as phosphine-susceptible, based on the knockdown method used. However, profound variations among species and populations were recorded. The species with the highest percentage of resistant populations was *R. dominica* (71.4% of the populations; resistance coefficient 0.5–4.1), followed by *S. oryzae* (57.1% of the populations; resistance coefficient 0.8–6.9), and *O. surinamensis* (9.5% of the populations; resistance coefficient 0.5–2.9). Regarding the intra-population variability in response to phosphine (slope of the knockdown time regression), the laboratory and slightly resistant populations of all species were homogenous, whereas the most resistant populations were strongly heterogeneous. Our data show that the occurrence of resistance in the Czech Republic is relatively widespread and covers a wide range of species, necessitating the need for the adoption of an action plan for resistance mitigation.

## 1. Introduction

Storage arthropods may invade and damage many types of stored commodities, both in developing and developed countries [1]. Apart from direct losses of stored grain caused by pests [2], the pest infestation of commodities and packaged foods [3] may result in their contamination with arthropod carcasses and allergens [4]. Currently, storage pest risks are elevated due to climatic changes [1,5], increasing both infestation of commodities during their international transport [6] and the resistance to many types of pesticides [2]. As a result, the effective control and resistance management of storage pests is a challenging issue for pest control operators, food industry managers, farmers, grain storekeepers, and commodity traders.

Fumigation with phosphine is by far the most frequently used chemical fumigation method used to control pests in stored products. Several commercial formulations are used for phosphine application, such as solid metal-phosphide formulations that release phosphine through a reaction with air moisture, or cylinderized phosphine, usually in combination with carbon dioxide or nitrogen [7,8]. Although the use of different commercial formulations varies according to marketing authorizations in different countries and geographical regions, phosphine is registered for post-harvest application worldwide, for a wide range of commodities and facilities. The activity of this gas was mainly dependent on its variable management practices, such as dose, exposure interval, sealing, physical conditions of the commodity, sorption, humidity, and temperature, as well as the target species and life stage [9,10,11,12]. The continuous and suboptimal use of phosphine, however, has led to the development of a decreased tolerance or even resistance by several major stored-product insect species, mainly beetles (Coleoptera), moths (Lepidoptera) and psocids (Psocoptera) [13], which is currently an important factor in choosing the right dose and exposure strategy. Continuous monitoring of resistance in different countries will allow legal local adaptations of dose-exposure fumigation protocols, as well as the assessment of the rate of increase in resistance in different species [13,14]. In this regard, early warning of the occurrence of resistance may help to establish preventive measures that will delay the spread of this phenomenon in wider geographical areas [13,15] through the transfer of highly infested commodities [6].

There are data on the occurrence and dynamics of phosphine resistance from different pest species originating from specific areas, such as Australia, China, India, Brazil, Africa, and the USA [13,16,17,18,19,20,21]. Recently, Sakka et al. [22] and Agrafioti et al. [14] reported that many of the populations of different stored-product beetle species that had originated from Greece and some other EU countries were found to be resistant to phosphine, but to a different degree. However, there is still limited information on the occurrence and degree of resistance of storage pests from various regions of Europe, especially in eastern and central Europe, despite the fact that these areas are major producers of durable commodities such as grains and legumes [22,23,24].

The basic evaluation protocol for quantifying the occurrence of resistance to phosphine is the Food and Agriculture Organization (FAO) method, which is based on exposures that are usually 20 h and concentrations that are usually approximately 30 ppm [14,16,17,25,26]. Despite the fact that the FAO protocol provided the first scientific series of data to estimate resistance, from the practical perspective it is considered as laborious and mostly only applicable under laboratory conditions [14]. As such, the FAO method cannot be easily operated by the fumigators, pest control operators, and advisors. Moreover, this method is mostly focused on area-wide surveillance of resistance development rather than an application-targeted commercial tool [22,27]. At the same time, FAO-related data may not always be easily transferable in “real world” fumigations, as they may not correspond to the actual resistance status of a given population at the time of the fumigation. Therefore, several generations of scientists have devoted significant effort to finding an easier alternative or complementary method for the evaluation of the occurrence and the quantification of resistance in commercial applications [13,14,19,27,28]. As a result, there are several modified versions of the FAO method that have been suggested for this purpose, as well as alternative rapid diagnostic tests that are sufficiently different from a fixed dose–exposure combination [27,28,29,30,31,32,33,34]. One of them is commercially available as a ready-to-use kit under the trade name Detia Degesch Phosphine Tolerance Test Kit, which was later renamed as Phosphine Tolerance Test (PTT, Detia Degesch GmbH, Laudenbach, Germany) [27,35,36,37]. Commercial availability of this kit enables a high level of standardization and thus a good direct comparison of data obtained by various studies at a global scale. This evaluation is based on short exposures, usually <15 min, to elevated concentrations, usually 3000 ppm, and it provides predictions of insects’ tolerance to phosphine according to their “speed to immobilization” [27]. Hence, PTT is based on a fixed concentration, but at the same time offers a “plasticity” in the exposure interval suggested, in contrast with the rest of the “dose bioassay” protocols that have specific exposure intervals at different concentrations [14,27]. Moreover, it seems that the immobilization data under short exposures to phosphine correlate well with predicted mortality, and thus, the predicted susceptibility to phosphine [14,22]. Indicatively, Agrafioti et al. [14] compared PTT with the FAO protocol and demonstrated a very good correlation for the vast majority of the species and populations tested, suggesting that longer exposure protocols that are based on mortality can be substituted by shorter ones that are based on immobilization. Finally, just like PTT is able to quantify susceptibility among populations, populations with different mobility patterns may react in a different way to PTT. Nevertheless, this hypothesis must be further examined with additional populations and treatment scenarios from a wider range of geographical areas.

Considering the lack of information on resistance to phosphine from most parts of Europe and taking into account the need for further testing of PTT with a wider range of populations, we have carried out a series of tests with populations that have been sampled from the Czech Republic. This sampling resulted in the collection of populations of the rice weevil, *Sitophilus oryzae* (L.) (Coleoptera: Curculionidae), the saw-toothed grain beetle, *Oryzaephilus surinamensis* (L.) (Coleoptera: Silvanidae), and the lesser grain borer, *Rhyzopertha dominica* (F.) (Coleoptera: Bostrychidae)—species that are considered as major pests in different types of facilities and commodities at a global scale, including the European Union (EU) [5,38].

## 2. Materials and Methods

### 2.1. Tested Insects and Populations

The field populations of the three species above were collected from Czech grain stores following the same methodology as the previous survey of arthropod fauna in Czech storage facilities [38]. The samples came from farm stores in Bohemia and Moravia, which are agricultural wheat and barley growing regions of the Czech Republic. After sampling, the insects were reared on different diets, which were wheat kernels for *S. oryzae* and *R. dominica* and ground wheat, oat flakes, and yeast (ratio 5:5:1) for *O. surinamensis*. All species were kept in incubators set at 25 ± 1 °C, 60–65% relative humidity (RH), and continuous darkness. For the resistance tests we used adults of the F3 generation after sampling, which were 7 to 14 days old. The standard laboratory Crop Research Institute (CRI) populations of each species were used as “controls”, i.e., susceptible to phosphine. They were collected at organic farms and maintained at the CRI laboratory at the same conditions as above for at least 15 years. For the purpose of the current survey, we collected 21 field populations of *S. oryzae*, 21 populations of *O. surinamensis*, and 15 populations of *R. dominica* from the Czech farm grain stores.

### 2.2. Laboratory Resistance Tests and Statistics

The sensitivity of various species and strains to phosphine was estimated by the PTT adapted by Steuerwald et al. [35], based on a method originally suggested by Reichmuth [29]. The PTT test is based on the simple rule that insects that are still moving after a certain predefined time interval of exposure to 3000 ppm of phosphine are considered tolerant/resistant to phosphine, and hence, the indicator of reduced susceptibility is insect immobilization, not mortality [27,35]. In the current experiment, we have used the revised knockdown time intervals for the three species, which were 10, 14 and 15 min for *S. oryzae*, *O. surinamensis*, and *R. dominica*, respectively, as suggested by Athanassiou et al. [27], and have now been incorporated in the PTT instructions and label [36,37]. The coefficient resistance was simply expressed as a ratio of the estimated KT_99_ or KT_100_ to the knockdown time intervals suggested for susceptible populations in the updated protocols of the commercial PTT kits [27].

The PTT contains a canister of 5 lt in capacity, on which the gas is generated through tablets and a syringe of 100 mL, which is used as the “exposure chamber” of the insects at a fixed concentration (set at 3000 ppm). The PPT requires phosphine concentration estimation using high-precision Dräger tubes Phosphine 25/A (Dräger Safety, Hamburg, Germany). More details regarding the utilization of PTT at the laboratory scale can be found in the studies of Agrafioti et al. [14] and Athanassiou et al. [27]. In our tests, within each syringe we placed 10 adults per species and population, and the entire procedure was repeated 20 times, with different sets of syringes for each combination. Then, the time to immobilization, also known as the knockdown time, was recorded visually.

The knockdown time 99% (KT_99_) per species and population was analyzed through a logistic regression knockdown model (χ^2^ test) using the statistical program XLSTAT (Addinsoft, France). The values of knockdown time to reach 100% (KT_100_) were calculated as the average values from all replications. The KT_100_ values were compared among the populations of each species by using one-way ANOVA with the package STATISTICA 12 (StatSoft CR s.r.o.). The KT_100_ values were separated by post hoc Tukey’s HSD test. Two resistance coefficients were estimated separately: (i) the KT_100_ parameter (calculated according to the PTT time); (ii) the KT_99_ parameter (calculated by regression model). The resistance coefficient for KT_100_ was established as the ratio of KT_100_ for the Czech field-collected strains to the fixed KT_100_ value reported as the resistance threshold level in the PTT protocol (i.e., fixed value KT_100_ ≤ 14 min for sensitive strains of *O. surinamensis,* KT_100_ ≤ 15 min for those of *R. dominica*, and KT_100_ ≤ 10 min for those of *S. oryzae*). On the other hand, the resistance coefficient for KT_99_ was estimated as the ratio of the KT_99_ of Czech field strains to the KT_99_ of the CRI-susceptible reference laboratory strains (i.e., *O. surinamensis* CRI–OsLab–KT_99_ = 7.94, *S. oryzae* CRI–SgLab–KT_99_ = 7.90, and *R. dominica* CRI–RdLab–KT_99_ = 8.79; see data presented in Table 1, Table 2 and Table 3). The time course of the knockdown effect at each exposure time was evaluated as a percentage of the average value of the knockdown effect achieved by the adults in 20 replications for each exposure time.

Apart from the above-described statistic KT-based logistic regression, we followed graphical data that are used for presentation and interpretation in mosquito studies [39,40]. These studies suggest graphical plotting of No. (or %) of knocked down individuals against time for each of the evaluated populations. The pattern shape of each curve gives a rapid visual indication of how field populations respond to an identical concentration of a toxic agent. When all individuals from the tested population react in a similar way, their dose–response curve is relatively steep, and the population is considered homogenous in terms of its sensitivity/tolerance. On the other hand, if some individuals of the population are much more tolerant than others, then the dose–response curve is flatter and the population is considered heterogeneous. Homogenous populations differing in insecticide resistance/sensitivity have a similar shape, but their base is shifted in parallel on the *x*-axis to the right-hand direction, while the larger the distance from the sensitive reference laboratory population, the higher the resistance of the compared field population.

## 3. Results

The results of the logistic regression model of phosphine for knockdown time (KT) and ANOVA evaluation of KT_100_ (based on the PTT protocol) or KT_95_ are summarized in Table 1, Table 2 and Table 3. Significant differences in the KT_100_ and KT_95_ values were recorded among the tested strains for *S. oryzae* (Table 1: KT_100_-F = 143.5; df = 11; *p* < 0.001), *O. surinamensis* (Table 2: KT_100_-F = 636.8; df = 10; *p* < 0.001), and *R. dominica* (Table 3: KT_100_-F = 16.7; df = 6; *p* < 0.001).

Although the survey revealed resistant populations, 57.1% of the tested field populations were classified as susceptible to phosphine. The highest number of the resistant populations were found in *R. dominica* (71.4%), followed by *S. oryzae* (57.1%). In contrast, only two populations (9.5%) of *O. surinamensis* showed some evidence of resistance. The level of resistance, measured as a coefficient of resistance, differed among the tested species (Table 1, Table 2 and Table 3). The broadest range of coefficient resistance values was recorded for *S. oryzae* (from 0.8 to 6.9×), followed by *R. dominica* (from 0.5 to 4.1×), while the values of the resistance coefficients for *O. surinamensis* were much narrower (from 0.5 to 2.9×). In fact, several field populations of *O. surinamensis* were found to have even lower coefficients of resistance than the laboratory strain, which is indicative of their susceptibility to phosphine.

**Figure 1:**
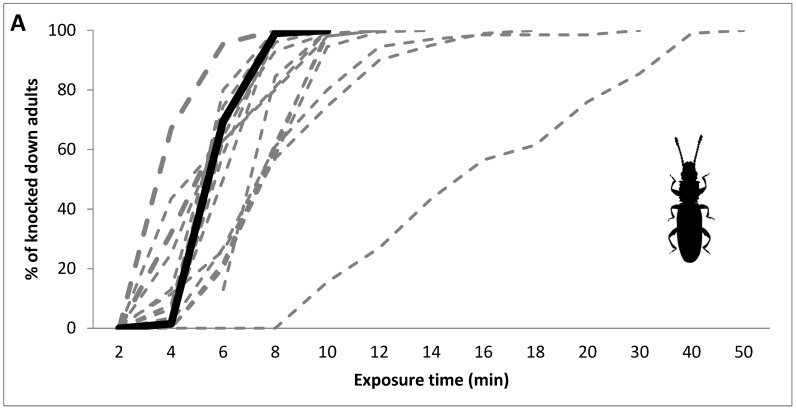

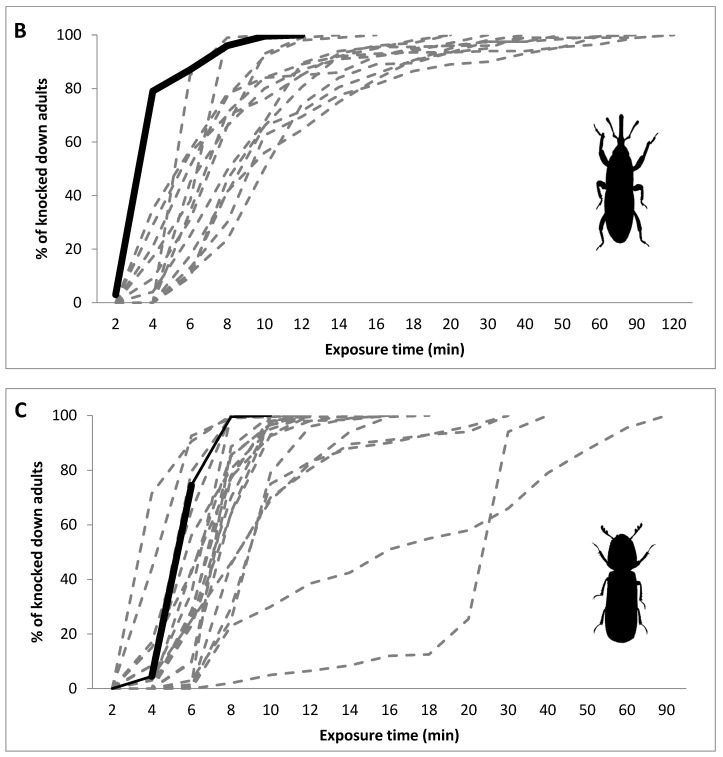
(**A**–**C**) Percentage of knocked down adults of different populations of the three tested species of stored-product pests (**A**—*O. surinamensis*; **B**—*S. oryzae*; **C**—*R. dominica*) after exposure to phosphine at 3000 ppm for different observation intervals (in minutes). The black, solid line shows the benchmark values for the laboratory population; the grey, dashed lines show the values for the field populations.

Figure 1 shows the time course of the knockdown effect (expressed as %) and homogeneity of the laboratory and field populations of the tested species during exposure to 3000 ppm of phosphine, according to the PPT protocol. The larger gaps between the curves of the field population and the reference susceptible laboratory population were mainly observed in *R. dominica* and *S. oryzae*, which is indicative of the higher resistance level as compared to the respective figures for *O. surinamensis*. In contrast, for the latter species, with one exception, most of the curves of field populations were relatively closely aggregated (from both sides) around the reference curve of the sensitive strain. Regarding the intra-population variability in response to phosphine, as characterized by the flat/steep shape and slope of the knockdown time regression, the laboratory and slightly resistant populations of all species were homogenous (steep curves), whereas the most resistant populations were strongly inhomogeneous (flat-shaped curves) in all of the tested species.

## 4. Discussion

In the last few years, there has been an increased amount of data for the occurrence of phosphine resistance in many species, which clearly indicates that this phenomenon is extremely widespread [13]. The recently published works from Europe have started to accumulate documentation that phosphine resistance can be found on this continent, possibly at a lower frequency as compared with other areas [14,22,23,24,41]. The current work can be considered as a continuation of this series of publications, providing additional data for central and northern Europe. The data reported here clearly show the occurrence of populations that are resistant to phosphine in the Czech Republic, especially in the case of *R. dominica* and *S. oryzae*, which are the primary colonizers of sound grain kernels [2,42] and related cereal materials [43]. The geographical location of the Czech Republic, which is in the center of Europe, may have an additional implication in area-wide resistance management strategies, as this country has an important role as a transit area of grains to other countries, through trucks, railcars, river boats etc. At the same time, the in-transit fumigation to control insects in bulked grains during transportation may not be effective due to the fact that the traditional means of transport may be leaky and unable to maintain sufficient concentrations of phosphine for long intervals [11,28,44]. For instance, in a series of tests in different storage structures, Agrafioti et al. [28] found that grain fumigations in ship holds were not effective, and phosphine concentrations were not sufficient to kill the exposed insects.

The actual risk level depends on the extent and incidence/frequency of resistance which may vary substantially among pest species and populations. At the same time, considerable variations may occur in the level of resistance, as previous studies documented that various species of stored-product pests have substantially different levels of resistance, i.e., strong vs. weak resistance [13,45], and frequency of resistance in various countries and geographical regions [13,14,16]. The current general geographical patterns of phosphine resistance indicate that some species of stored-product beetles tend to independently develop resistant field populations in many geographical areas [13], whereas some other pest species, such as the granary weevil, *Sitophilus granarius* (L.) (Coleoptera: Curculionidae) [14,24,41,46], still tend to be resistant to a much lesser extent.

The tested Czech populations of *O. surinamensis* have a relatively lower frequency and level of resistance than the populations of the other two species, which could be attributed to the higher prevalence of *S. oryzae* and *R. dominica* in raw grains as compared to the respected prevalence of *O. surinamensis*. Thus, in bulked grains, *O. surinamensis* populations are likely to be exposed fewer times to phosphine fumigations as compared with the other two species. For instance, in a surveillance in bulked grains in central Greece, Athanassiou and Buchelos [47] found that *R. dominica* and *S. oryzae* were more numerous than *O. surinamensis* in grain trier samples. Moreover, *R. dominica* and *S. oryzae* are internal feeders and, as such, their immature life stages are less affected by some of the currently used insecticides [2]. Information on these two species shows a relatively high incidence of phosphine resistant populations in large-scale geographical surveys [13]. For those two species there is also a solid documentation on the genetic and molecular background of resistance to phosphine [45,48,49,50].

In a recent work, Gautam et al. [51] reported that even though *O. surinamensis* is one of the key stored-product pests worldwide, publications of phosphine resistance in *O. surinamensis* are scarce, in contrast with many other storage key pests, but populations of this species that are resistant to phosphine occur in large geographical zones in the USA. The extensive literature review of Nayak et al. [13] on the topic shows mainly isolated reports on *O. surinamensis* resistance from geographically distant locations. The earliest records of the resistant populations of this species likely originate from Australia [52,53] (Herron, 1990; Emery, 1994). Nevertheless, there are areas where the frequency of resistance to phosphine of different populations of *O. surinamensis* is extremely high, as in the case of Brazil [54,55,56,57] and Thailand [58]. The most recent records of *O. surinamensis* resistance are from the USA [51,59], Greece [14], and Turkey [41]. However, even in these cases, the frequency of resistance to phosphine for this species is not higher than that of other major stored-product beetle species, including *S. oryzae* and *R. dominica* [14,41]. The survey by Agrafioti et al. [14] from Greece revealed that although all tested *R. dominica* populations were resistant, resistance for *O. surinamensis* was recorded only in about 50% of the populations tested. Bioassays conducted in Turkey by Kocak et al. [41] showed that resistance was frequently found in the sampled populations of *S. oryzae* (39.3%), ranging from 3–200 fold, whereas resistant populations of *O. surinamensis* indicated a much lower proportion (18.7%). Nevertheless, the range of resistance level was much narrower and higher in *O. surinamensis* (from 389× to 459×) than that of *S. oryzae* [41]. So far, we have no simple explanation for the difference, except that *O. surinamensis* may infest different types of frequently fumigated commodities, such as dried fruits, in Turkey [60] or Greece [44], as compared with grains that are mainly stored and fumigated in the Czech Republic.

Our findings have several practical implications in terms of commercial fumigations regarding resistance management strategies. These strategies can involve rotation of different active ingredients, such as contact insecticides, or even non-chemical methods, such as aeration, or controlled/modified atmospheres [2,61,62,63]. In fumigation itself, an important component is the compliance with best management practices, in order to avoid any suboptimal routines or malpractice [9,64,65]. However, the knowledge obtained so far clearly shows that most of the fumigation failures, in terms of complete insect control, are due to the absence of good fumigation practices, such as the occurrence of leaky structures and the application of low concentrations, and not due to resistance [24,28,66].

To ensure that labels and fumigation protocols are followed and ensured properly, phosphine concentrations need to be measured and documented during and after the fumigation, for both workplace safety issues [67] and to secure phosphine efficacy against all species, populations, and life stages [11,23,28]. As the labels of many phosphine-releasing products legally offer a certain range of doses and exposure times, so the concentration can be, up to a certain degree, adjusted, according to the application scenario and the susceptibility level of the target species [44,68]. For this purpose, the adaptation of a rapid diagnostic that can be easily utilized towards this direction is essential to guide possible adjustments in the fumigation practices.

## 5. Conclusions

The presented phosphine resistance survey in this publication included 56 field populations classified in three stored-product beetle species, collected in Czech farm grain stores. It was found that the tested Czech populations of *O. surinamensis* have a relatively lower frequency and level of resistance than the populations of *S. oryzae* and *R. dominica*. The results show that the occurrence of resistance in the Czech Republic is widespread and includes economically important species, highlighting the need for fumigation alternatives [61,66,69,70,71] as well as the establishment of integrated resistance management programs in grain stores [2].

## Figures and Tables

**Table 1:** Susceptibility or tolerance of 22 populations of *Sitophilus oryzae* to phosphine based on the knockdown rapid test (PTT–Degesch, Gemany) from the Czech grain stores and the statistical comparison among the tested populations (21 field populations and 1 reference laboratory SoLab population). (Different letters (a–e) indicate statistically significant differences; *n* = numbers; *p* = *p*-value).

Population	*n*	Susceptibility Time Period (min)	KT_100_ Average (min)	Resistance Coefficient KT_100_	Slope ± SE	KT_99_ (95% CI)	Resistance Coefficient KT_99_	χ^2^	df	*p*
**SoLab**	**20**	**10**	**8.1 ± 0.1 a**	**0.8**	**13.72 ± 3.99**	**7.90 (6.73–13.07)**	**1.0**	**52.99**	**1**	**<0.0001**
SoRnk	20	10	7.6 ± 0.3 a	0.8	8.54 ± 2.59	7.81 (6.19–17.32)	0.7	49.56	1	<0.0001
SoBor	20	10	7.6 ± 0.2 a	0.8	7.80 ± 2.25	7.25 (5.65–15.57)	0.7	32.01	1	<0.0001
SoSml	20	10	8.0 ± 0.0 a	0.8	9.64 ± 2.79	9.03 (7.25–18.42)	0.9	31.97	1	<0.0001
SoMil	20	10	8.0 ± 0.0 a	0.8	14.16 ± 4.63	8.90 (7.54–17.46)	0.9	33.82	1	<0.0001
SoZb3	20	10	8.2 ± 0.2 a	0.8	11.08 ± 2.94	8.30 (6.88–13.92)	0.8	59.16	1	<0.0001
SoSlc	20	10	10.0 ± 0.0 ab	1.0	27.01 ± 9.08	8.81 (8.06–12.21)	8.8	57.71	1	<0.0001
SoTrn	20	10	10.1 ± 0.1 ab	1.0	10.78 ± 2.86	10.63 (8.89–17.57)	1.1	56.79	1	<0.0001
SoOur	20	10	10.3 ± 0.3 ab	1.0	7.49 ± 1.74	12.04 (9.55–21.08)	1.2	47.89	1	<0.0001
SoIns	20	10	10.3 ± 0.2 ab	1.0	14.56 ± 3.94	10.74 (9.32–16.25)	1.1	61.06	1	<0.0001
SoTuc	20	10	10.6 ± 0.4 ab	1.1	10.53 ± 2.57	11.31 (9.50–17.52)	1.1	87.02	1	<0.0001
SoKov	20	10	10.6 ± 0.2 ab	1.1	12.38 ± 3.41	10.36 (8.81–16.71)	1.0	60.10	1	<0.0001
SoT56	20	10	10.7 ± 0.3 ab	1.1	10.72 ± 2.65	11.57 (9.74–18.03)	1.2	68.10	1	<0.0001
SoKas	20	10	10.8 ± 0.2 ab	1.1	11.98 ± 2.67	11.98 (10.03–19.24)	1.2	53.58	1	<0.0001
SoT59	20	10	10.9 ± 0.2 ab	1.1	7.64 ± 1.79	11.97 (9.55–20.75)	1.2	48.34	1	<0.0001
SoSla	20	10	12.0 ± 0.5 ab	1.2	11.03 ± 2.62	11.99 (10.20–17.76)	1.2	80.48	1	<0.0001
SoCer	20	10	12.8 ± 0.3 ab	1.3	12.68 ± 2.95	13.43 (11.63–18.98)	1.3	81.90	1	<0.0001
SoUnl	20	10	14.4 ± 0.4 b	1.4	6.26 ± 1.18	18.88 (14.99–29.97)	1.9	66.39	1	<0.0001
So26	20	10	22.2 ± 1.7 c	2.2	6.23 ± 1.11	21.83 (17.60–32.92)	2.2	84.52	1	<0.0001
So27	20	10	22.4 ± 1.5 c	2.2	6.74 ± 1.20	21.00 (17.17–30.92)	2.1	88.67	1	<0.0001
So29	20	10	35.5 ± 1.1 d	3.6	7.04 ± 1.30	47.10 (36.05–81.30)	4.7	73.56	1	<0.0001
SoEip	20	10	69.0 ± 4.5 e	6.9	2.89 ± 0.43	111.17 (70.50–248.39)	11.1	77.57	1	<0.0001

**Table 2:** Sensitivity or resistance level of 22 populations of *Oryzaephilus surinamensis* to phosphine based on the knockdown rapid test (PTT–Degesch, Gemany) from the Czech grain stores and the statistical comparison among the tested populations (21 field populations and 1 reference laboratory OsLab population). (Different letters (a–f) indicate statistically significant differences; *n* = numbers; *p* = *p*-value).

Population	*n*	Susceptibility Time Period (min)	KT_100_ Average (min)	Resistance Coefficient KT_100_	Slope ± SE	KT_99_ (95% CI)	Resistance Coefficient KT_99_	χ^2^	df	*p*
**OsLab**	**20**	**14**	**8.1 ± 0.2 ab**	**0.6**	**15.04 ± 4.96**	**7.94 (6.83–14.34)**	**1.0**	**54.01**	**1**	**<0.0001**
OsBlo	20	14	6.8 ± 0.2 a	0.5	9.29 ± 3.22	6.54 (5.20–17.85)	0.5	35.07	1	<0.0001
Os37	20	14	8.0 ± 0.0 ab	0.6	12.26 ± 3.48	8.20 (6.86–14.17)	1.0	37.02	1	<0.0001
Os38	20	14	8.0 ± 0.1 ab	0.6	11.05 ± 3.04	8.32 (6.88–14.57)	1.0	48.31	1	<0.0001
OsOur	20	14	8.0 ± 0.0 ab	0.6	16.63 ± 6.75	7.96 (6.87–24.42)	1.0	38.83	1	<0.0001
OsMal	20	14	8.0 ± 0.0 ab	0.6	13.04 ± 4.00	8.72 (7.35–15.99)	0.6	34.95	1	<0.0001
OsDuj	20	14	8.0 ± 0.0 ab	0.6	7.77 ± 2.35	9.54 (7.32–24.30)	0.7	28.89	1	<0.0001
OsKas	20	14	8.0 ± 0.0 ab	0.6	18.22 ± 6.98	7.23 (6.35–14.93)	0.5	43.94	1	<0.0001
OsZb2	20	14	8.2 ± 0.1 ab	0.6	12.32 ± 3.42	8.28 (6.96–13.80)	1.0	50.72	1	<0.0001
OsRcp	20	14	8.3± 0.2 abc	0.6	16.61 ± 6.66	7.84 (6.80–21.31)	0.6	54.78	1	<0.0001
OsZb1	20	14	8.6 ± 0.2 abc	0.6	13.39 ± 4.06	8.53 (7.27–14.65)	0.6	51.53	1	<0.0001
OsChr	20	14	9.3 ± 0.3 bcd	0.7	7.40 ± 1.85	10.41 (8.19–19.38)	1.3	48.15	1	<0.0001
OsKuc	20	14	9.8 ± 0.1 bcd	0.7	12.33 ± 3.55	11.19 (9.42–19.55)	0.8	40.39	1	<0.0001
OsPls	20	14	10.0 ± 0.0 bcd	0.7	12.57 ± 3.61	7.56 (6.33–13.18)	0.5	39.00	1	<0.0001
OsPro	20	14	10.0 ± 0.0 bcd	0.7	5.33 ± 1.34	13.23 (9.52–31.89)	1.7	29.20	1	<0.0001
OsCho	20	14	10.4 ± 0.2 cd	0.7	9.57 ± 2.41	10.60 (8.76–17.45)	1.3	54.68	1	<0.0001
OsPol	20	14	10.4 ± 0.2 cd	0.7	11.73 ± 3.08	11.36 (9.61–18.17)	0.8	56.41	1	<0.0001
OsSla	20	14	10.9 ± 0.3 d	0.8	10.79 ± 2.64	12.06 (10.16–18.70)	1.5	67.28	1	<0.0001
OsBL1	20	14	13.5 ± 0.5 e	1.0	8.76 ± 1.81	14.55 (12.15–21.37)	1.0	82.49	1	<0.0001
OsBus	20	14	14.4 ± 0.4 e	1.0	5.52 ± 1.02	19.05 (14.77–31.40)	2.4	62.25	1	<0.0001
OsBur	20	14	15.5 ± 1.4 e	1.1	6.83 ± 1.29	16.24 (13.16–24.57)	1.2	88.70	1	<0.0001
OsIt2	20	14	40.0 ± 1.0 f	2.9	5.50 ± 0.96	41.89 (31.73–72.04)	5.3	93.96	1	<0.0001

**Table 3:** Sensitivity or resistance level of 15 populations of *Rhyzopertha dominica* to phosphine based on the knockdown rapid test (PTT–Degesch, Gemany) from the Czech grain stores and the statistical comparison among the tested populations (14 field populations and 1 reference laboratory RdLab population). (Different letters (a–f) indicate statistically significant differences; n = numbers; *p* = *p*-value).

Population	*n*	Susceptibility Time Period (min)	KT_100_ Average (min)	Resistance Coefficient KT_100_	Slope ± SE	KT_99_ (95% CI)	Resistance Coefficient KT_99_	χ^2^	df	*p*
**RdLab**	**20**	**15**	**8.7 ± 0.3 a**	**0.6**	**5.78 ± 1.36**	**8.79 (6.57–17.40)**	**1.0**	**37.40**	**1**	**<0.0001**
RdKas	20	15	8.0 ± 0.0 a	0.5	8.54 ± 2.53	10.59 (8.19–25.70)	0.7	26.72	1	<0.0001
RdMis	20	15	8.1 ± 0.1 a	0.5	18.70 ± 7.29	7.15 (6.31–14.54)	0.5	58.19	1	<0.0001
RdPel	20	15	11.8 ± 0.4 ab	0.8	5.92 ± 1.22	14.27 (11.07–24.58)	1.0	57.23	1	<0.0001
RdSml	20	15	11.4 ± 0.3 ab	0.8	7.53 ± 1.61	13.58 (10.91–22.08)	0.9	57.25	1	<0.0001
RdChc	20	15	16.5 ± 0.5 abc	1.1	3.92 ± 0.71	21.79 (15.81–40.41)	1.5	50.30	1	<0.0001
RdZer	20	15	16.5 ± 0.6 abc	1.1	5.86 ± 1.08	18.11 (14.38–28.30)	1.2	73.00	1	<0.0001
RdBno	20	15	22.4 ± 1.3 abcd	1.5	3.52 ± 0.64	27.99 (19.50–57.06)	1.9	44.83	1	<0.0001
RdAus	20	15	23.7 ± 3.7 abcd	1.6	4.74 ± 0.79	24.62 (18.88–40.04)	1.6	87.11	1	<0.0001
RdPod	20	15	26.0 ± 1.9 bcd	1.7	5.01 ± 0.87	28.76 (22.00–48.04)	1.9	80.97	1	<0.0001
RdBu2	20	15	27.6 ± 3.0 bcd	1.8	5.03 ± 0.86	25.55 (19.78–41.11)	1.7	82.92	1	<0.0001
RdOur	20	15	33.0 ± 4.3 cde	2.2	4.38 ± 0.73	31.84 (23.85–54.24)	2.1	100.24	1	<0.0001
RdCer	20	15	34.5 ± 7.3 de	2.3	3.82 ± 0.62	28.77 (21.08–50.33)	1.9	90,25	1	<0.0001
RdKlo	20	15	44.3 ± 4.8 e	3.0	4.05 ± 0.64	38.00 (27.96–65.65)	2.5	99.22	1	<0.0001
RdBus	20	15	61.4 ± 7.0 f	4.1	3.29 ± 0.50	51.66 (35.92–97.93)	3.4	93.49	1	<0.0001

## Data Availability

The data presented in this study are available in the manuscript.
